# Characterization of *Staphylococcus aureus* Isolates From Cases of Clinical Bovine Mastitis on Large-Scale Chinese Dairy Farms

**DOI:** 10.3389/fvets.2020.580129

**Published:** 2020-12-07

**Authors:** Kangjun Liu, Luyao Tao, Jianji Li, Li Fang, Luying Cui, Jun Li, Xia Meng, Guoqiang Zhu, Chongliang Bi, Heng Wang

**Affiliations:** ^1^College of Veterinary Medicine, Yangzhou University, Yangzhou, China; ^2^Jiangsu Co-innovation Center for Prevention and Control of Important Animal Infectious Diseases and Zoonoses, Yangzhou, China; ^3^Joint International Research Laboratory of Agriculture and Agri-Product Safety, The Ministry of Education of China, Yangzhou, China; ^4^College of Agriculture and Forestry Science, Linyi University, Linyi, China

**Keywords:** *Staphylococcus aureus*, bovine mastitis, MALDI TOF MS, molecular typing, virulence gene, antimicrobial resistance (AMR)

## Abstract

Bovine mastitis is a prevalent disease that causes serious economic problems globally in the dairy industry. *Staphylococcus aureus* is an important pathogen of bovine mastitis. This study was conducted to characterize *S. aureus* isolates from clinical bovine mastitis cases in large-scale dairy herds in China. *S. aureus* was isolated from 624 clinical mastitis cases and confirmed by matrix-assisted laser desorption ionization-time of flight mass spectrometry (MALDI-TOF MS). In total, 62 *S. aureus* isolates were obtained. Cluster analysis, genetic diversity, quantification of biofilm formation, antimicrobial resistance, and detection of virulence genes were performed on these isolates of *S. aureus*. Eight isolates harbored the *mecA* gene and were sensitive to oxacillin. MALDI-TOF MS cluster analysis revealed that the 62 isolates were divided into three major clusters (I, II, III) and eight main groups (A–H) at the distance level of 700. The *agr* II was the most prevalent (56.5%). The 62 *S. aureus* isolates were assigned to seven *spa* types. The most common *spa* type was t529(58.1%), followed by t2196 (14.5%), t518 (14.5%), t571(6.5%), t034 (3.2%), t2734 (1.6%), and t730 (1.6%). Five STs were identified from seven representative isolates as follows: ST630/CC8, ST97/CC97, ST50, ST398, and ST705. All isolates had the ability to form biofilm. Antimicrobial resistance was most frequently observed to ciprofloxacin (29%), followed by penicillin (24.2%), and streptomycin (9.6%). All isolates harbored the *fnbA, clfB* (100%), *icaA*, and *icaD* genes. This study provides the basis for the development of bovine mastitis prevention program on large-scale dairy farms.

## Introduction

Bovine mastitis, inflammation of the mammary gland, is predominantly induced by intramammary bacterial infection, and causes serious economic losses in the global dairy industry (1). *Staphylococcus aureus* (*S. aureus*) is one of the most important and common pathogenic microorganisms in bovine mastitis (2). *S. aureus* carries many virulence factors, such as hemolysins (Hla and Hlb), toxic shock syndrome toxin-1 (Tsst-1), leukocidin, fibronectin binding proteins (FnbA and FnbB), and clumping factors (ClfA and ClfB), which facilitate adhesion of *S. aureus* to the host extracellular matrix components, damaging host cells and impeding the immune system (3). *S. aureus* has developed antimicrobial resistance due to selective pressures from the indiscriminate use of antimicrobial agents (4), and multidrug-resistant (MDR) strains have emerged including methicillin-resistant *S. aureus* (MRSA). Furthermore, biofilm formation helps *S. aureus* survive in the pressure of antimicrobial agents and evade the host immune response, which can give rise to a persistent infection (5). Thus, mastitis caused by *S. aureus* has a low cure rate in cattle.

Conventional methods used for the epidemiological studies of *S. aureus* include pulsed-field gel electrophoresis (PFGE), multilocus sequence typing (MLST), polymorphism of protein A gene (*spa* typing), and accessory gene regulator typing (*agr* typing). Utilization of MALDI-TOF MS for identification and classification of species of the genus *Staphylococcus* has been evaluated in both human and veterinary medicine (6, 7). However, few studies have assessed the value of MALDI-TOF MS as an epidemiological typing tool in bovine mastitis. The MALDI-TOF MS fingerprinting approach could extend phenotypic and genotypic approaches, allowing for more detailed classification of *S. aureus*.

In China, large-scale dairy farms have developed rapidly in recent years and have become the mainstay of raw milk production. Although mastitis control programs have achieved considerable progress, the incidence of clinical mastitis remains high in large dairy herds in China (8). *S. aureus* infection can sometimes result in acute and clinical mastitis with changes in milk composition, leading to milk being discarded and even culling of cows. If appropriate control strategies are not taken in time to prevent the transmission of *S. aureus* in herds, immeasurable losses can occur on large-scale dairy farms. It is thus necessary to monitor the incidence of clinical mastitis caused by *S. aureus* on large-scale dairy farms. Moreover, data analysis regarding the epidemiology of *S. aureus* can provide references for developing scientific prevention and control programs. Currently, there is a lack of relevant data available for large-scale farms. The objective of this study was to provide epidemiological information on *S. aureus* isolates from large-scale Chinese dairy herds by examining cluster analysis, genetic diversity, biofilm formation, antimicrobial resistance, and virulence genes.

## Materials and Methods

### Samples

**Figure 1:**
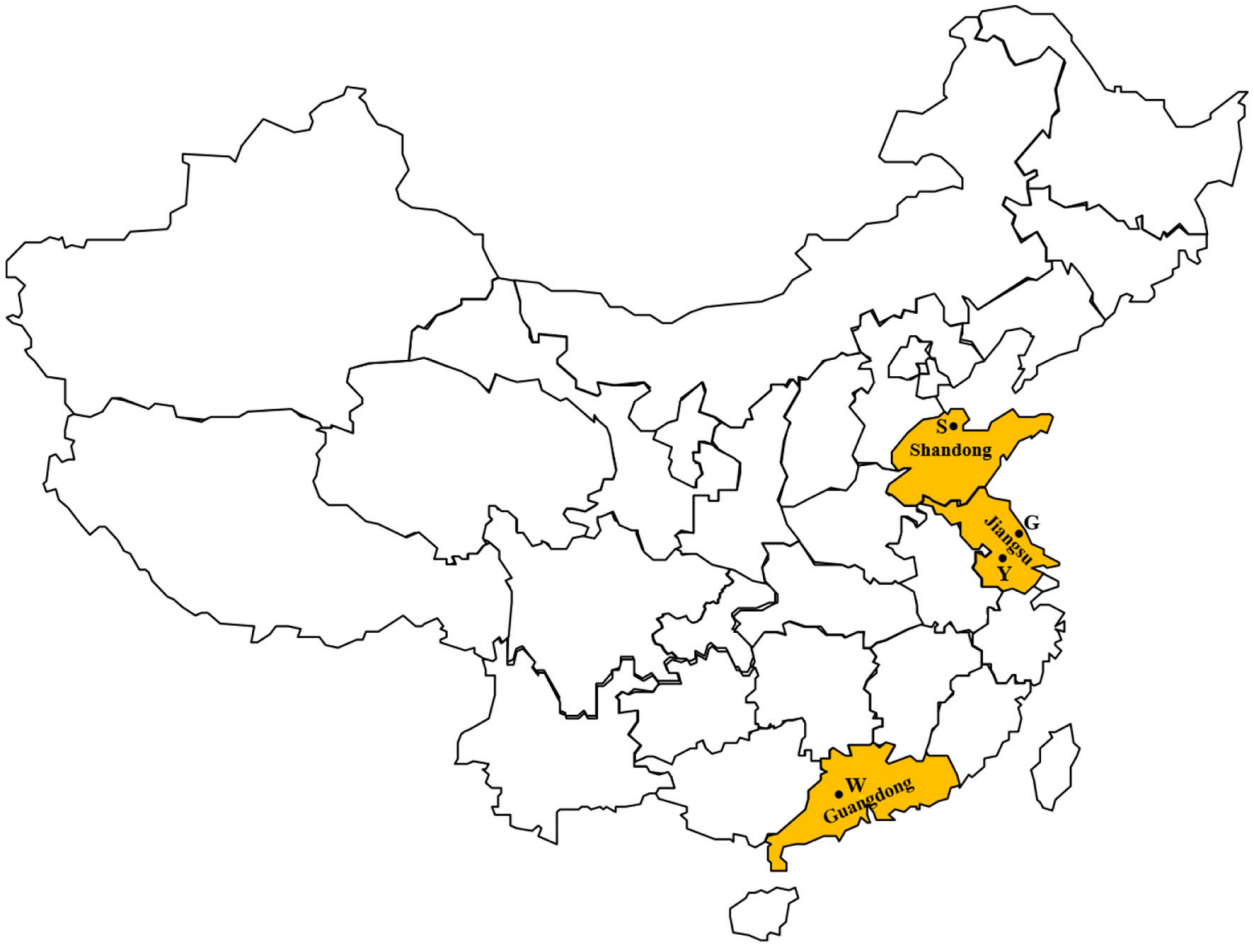
Geographic distribution of the farms in this study.

A total of 624 milk samples from cases of clinical bovine mastitis were collected aseptically from four farms between May 2016 and August 2017. These milk samples were collected from all the clinical mastitis cases during the investigation. Mastitis was initially identified by farm staffs at milking time, and was confirmed by a veterinarian. The four farms were in three provinces of China, including Shandong (*n* = *1*), Jiangsu (*n* = *2*), and Guangdong (*n* = *1*) (Figure 1). These provinces are located in the eastern coastal areas, and have distinctly different climates. Shandong province has a cool and dry climate, while it is warm and humid in Guangdong province. Jiangsu province is in a transitional zone between temperate and subtropical zones, and has a mild climate. According to a report of the Dairy Association of China, the average cow population of Chinese farms was 166 in 2019. In a previous investigation, farms in China with a cow population more than 500 were defined as large-scale farms (8). In this study, there were at least 1,000 lactating Holstein-Friesian cows on the farms, hence these were large-scale dairy farms. Related information on the four farms is shown in Table 1.

**Table 1:** The population size, milk yield, and antibiotics administration of the farms.

**Farm**	**Population size**	**Milk yield^**a**^ (kg)**	**Management of dry cow**	**Treatments of mastitis**
S	2,040	8,620	Cefalexin and kanamycin	Amoxicillin and clavulanate, and benzylpenicillin potassium
Y	1,000	7,900	Benzylpenicillin, nafcillin sodium, and dihydrostreptomycin sulfate	Gentamicin and amoxicillin
G	12,000	10,400	Benzylpenicillin, nafcillin sodium, and dihydrostreptomycin sulfate	Cefalexin and kanamycin
W	11,000	9,500	Benzylpenicillin, nafcillin sodium, and dihydrostreptomycin sulfate	Gentamicin, amoxicillin, and lincomycin

a *Milk yield is milk production/number of adult cows in 2017*.

### Isolation and Identification of *S. aureus*

Milk samples (100 μL) were inoculated on Baird-Parker agar (BPA) supplemented with 5% egg yolk and tellurite (Hope Biotech Co., Ltd, Qingdao, China), and cultured at 37°C for 24 h. *S. aureus* isolates were initially identified by morphology and biochemical methods (colony morphology, Gram staining, and catalase testing), then were confirmed by MALDI-TOF MS (Bruker Daltonics, Bremen, Germany). MRSA strains were confirmed by targeting the *mecA* gene and oxacillin disc diffusion test.

### Cluster Analyses of *S. aureus* Isolates

Cluster analyses of *S. aureus* isolates were conducted using MALDI Biotyper OC version 4.0.19 (Bruker Daltonics, Bremen, Germany).

### Molecular Typing

The *agr* types I–IV were determined by multiplex PCR assay as described previously (9). Briefly, a 25 μL reaction mixture was prepared containing 12.5 μL 2 × EasyTaq PCR SuperMIX (TransGen Biotech Co., Ltd, Beijing, China), 2 μL DNA, 1 μL each of forward and reverse primers (10 μmoL), and 8.5 μL ultrapure water. The thermal cycling program comprised an initial denaturation at 94°C for 3 min, followed by 30 cycles of denaturation at 94°C for 30 s, annealing at 55°C for 30 s, and extension at 72°C for 30 s, and a final extension at 72°C for 5 min. The polymorphic X region of the *spa* gene was amplified by the method described previously (1). MLST was performed as described by Enright et al. and the seven housekeeping genes were amplified by PCR (10). PCR amplicons were submitted to Tsingke Biological Technology Co., Ltd (Nanjing, China) for sequencing. The *spa* types were determined using the *spa*-server (http://spa.ridom.de/;) (11). The allelic profile of each strain was identified and assigned to the respective sequence type (ST) using the PubMLST database (https://pubmlst.org).

### Biofilm Formation

Quantification of biofilm was performed by spectrophotometry in microplates using crystal violet staining as described previously (12). Isolates were classified into the following categories: no biofilm producer, weak, moderate, or strong biofilm producer according to a previous method (13).

### Antimicrobial Susceptibility Tests

Antimicrobial susceptibility of *S. aureus* isolates was determined by the agar disk diffusion method according to the guideline of the Clinical Laboratory Standards Institute (14). Antimicrobial agents often used on farms to treat bovine mastitis were selected and included the following: penicillin (P, 10 U), cephalexin (CEP, 30 μg), ceftiofur (EFT, 30 μg), gentamicin (CN, 10 μg), streptomycin (STR, 10 μg), clindamycin (DA, 2 μg), ciprofloxacin (CIP, 5 μg), doxycycline (DX, 30 μg), tetracycline (TE, 30 μg), amikacin (AK, 30 μg), and kanamycin (KAN, 30 μg). All antimicrobial agents were purchased from Hangzhou Microbial Reagent Co., Ltd., except ceftiofur (Oxoid). MDR isolates were defined as showing resistance to three or more antimicrobial agents (4).

### Detection of Virulence Determinants

Virulence genes, including *lukM, fnbA, fnbB, clfA, clfB, hl*α, *hl*β, *icaA, icaD, pvl, bap*, and *tsst-1* were detected by PCR as described previously (15–21). Positive and negative controls were included in all PCRs.

### Statistical Analysis

Statistical analysis was performed with SPSS Statistics 22.0 software (IBM, USA). Chi-square tests were used to analyze the association between multidrug resistance and biofilm formation ability. A *p*-value of < 0.05 was deemed to be statistically significant.

## Results

### Incidence of *S. aureus* and MRSA

The incidence of clinical mastitis was 14.8, 5.5, 1.1, and 1.3% on farms S, Y, G, and W, respectively. From the milk samples, 62 *S. aureus* isolates were obtained, including 18 isolates in 301 Shandong samples, 39 isolates in 183 Jiangsu samples, and 5 isolates in 140 Guangdong samples. The proportion of clinical mastitis caused by *S. aureus* was 6, 14.5, 24.2, and 3.6% on farm S, Y, G, and W, respectively. Eight isolates of *S. aureus* from farm Y harbored the *mecA* gene and were sensitive to oxacillin, hence were classed as oxacillin-susceptible and *mecA*-positive strains (OS-MRSA).

### Cluster Analyses of *S. aureus* Isolates

**Figure 2:**
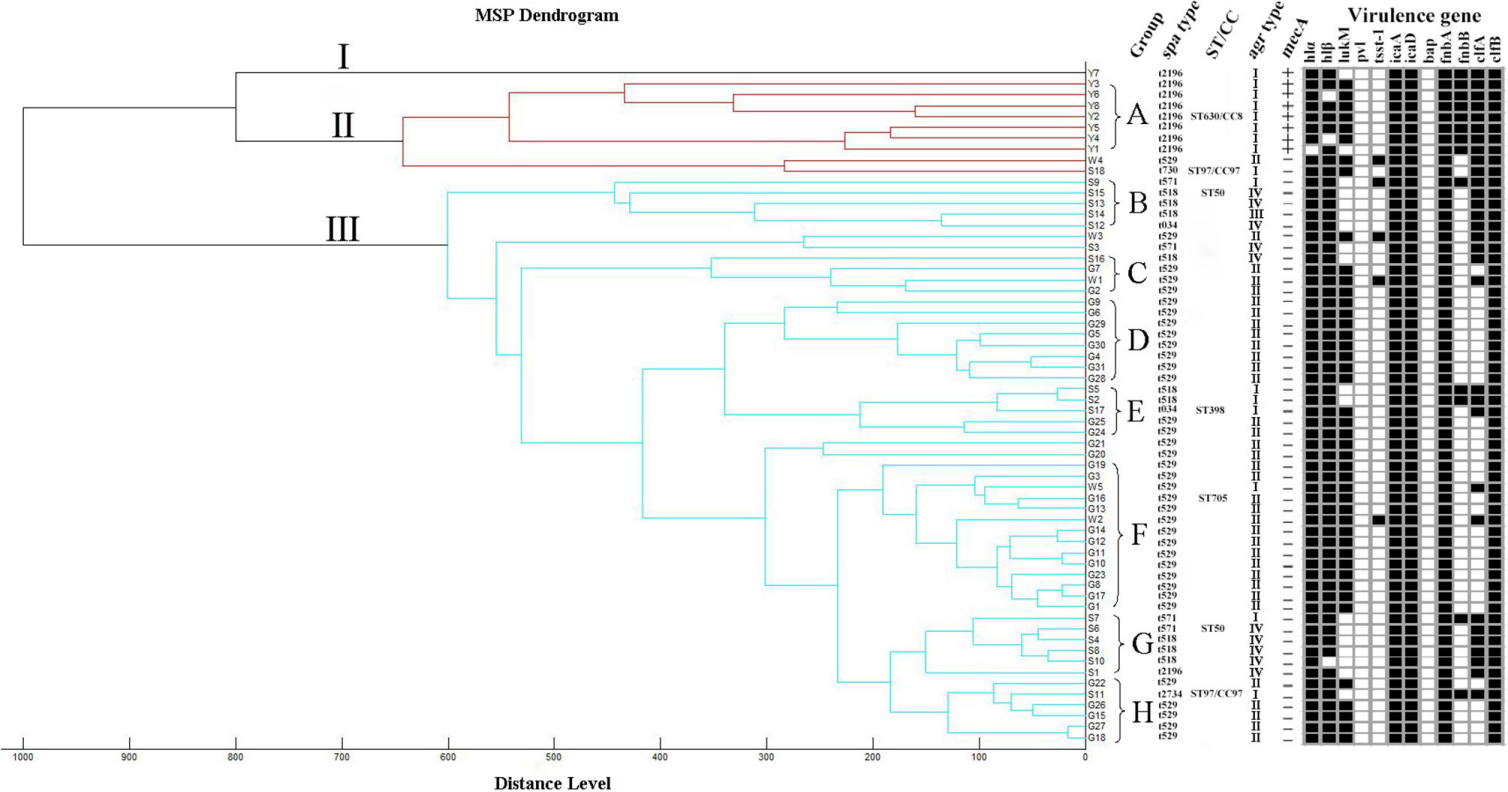
Dendrogram and *spa* types, ST/CC, *agr* types, *mecA* gene, and virulence genes. MSP dendrogram generated by the standard MALDI Biotyper MSP creation method for 62 *S. aureus* isolates. The scale below the dendrogram indicates the relative distance. Letters **(A–H)** represent 8 groups. The detection of virulence genes was summarized by a heat map. Black squares denote that the studied genes were detected. White squares denote that those isolates lack these genes.

A dendrogram was generated based on the protein spectral fingerprints of the 62 *S. aureus* isolates (Figure 2). This MALDI-TOF MS cluster analysis showed that the 62 isolates were divided into three major clusters (I, II, III) and eight main groups (A–H) at the distance level of 700. The majority of isolates (83.9%, 52/62) were classified into cluster III. Cluster I and II contained 1 and 9 isolates, respectively. In groups F and H, the isolates obtained from different farms were classified into sub-clades at a close relative distance (<200). Eight *mecA*-positive *S. aureus* strains were discriminated from the other 54 methicillin-sensitive *S. aureus* (MSSA) and were grouped in clusters I and II.

### Molecular Typing

The *agr* alleles were successfully identified in the 62 isolates by multiplex PCR. As shown in Table 2, *agr* II was most prevalent (56.5%; 35/62), followed by *agr* I (24.2%; 15/62), *agr* IV (17.7%; 11/62), and *agr* III (1.6%; 1/62). The most common *agr* type in *S. aureus* isolated from Shandong herd was *agr* IV, whereas, the *agr* II was predominant in *S. aureus* isolates obtained from herds in Jiangsu and Guangdong provinces. The 62 *S. aureus* isolates were also assigned to seven *spa* types. The predominant *spa* type was t529 (58.1%; 36/62), followed by t2196 (14.5%; 9/62), t518 (14.5%; 9/62), t571 (6.5%; 4/62), t034 (3.2%; 2/62), t2734 (1.6%, 1/62), and t730 (1.6%, 1/62) (Figure 2). The *spa* type t529 was found in *S. aureus* isolated from farms G and W. Six *spa* types (t2196, t571, t2734, t034, t730, and t518) were found in *S. aureus* isolated from farm S. Seven representative isolates that belonged to different *spa* types were then subjected to MLST. Five sequence types (STs) were identified as follows: ST630/CC8, ST97/CC97, ST50, ST398, and ST705. The corresponding relationship between STs and *spa* types is shown in Table 3.

**Table 2:** The *agr* types and biofilm formation of 62 *S. aureus* isolates.

**Origin**	**No. of strains**	***agr type***				**Percentage (no.) of** ***S. aureus*** **with biofilm production**		
		**I**	**II**	**III**	**IV**	**Weak**	**Moderate**	**Strong**
Shandong	18	38.8%	0	5.6%	55.6%	27.8%	38.9%	33.3%
Jiangsu	39	20.5%	79.5%	0	0	79.5%	7.7%	12.8%
Guangdong	5	20%	80%	0	0	20%	80%	0
Total	62	24.2%	56.5%	1.6%	17.7%	59.7%	22.6%	17.7%

**Table 3:** The *spa* types and STs of 7 representative isolates.

**Representative isolates**	***spa* types**	***spa* repeats**	**STs**
G16	T529	r04r34	ST705
Y2	T2196	r04r34r22r25	ST630
S6	T571	r08r16r02r25r02r25r34r25	ST50
S11	T2734	r07r23r21r17r34r34r33r34	ST97
S17	T034	r08r16r02r25r02r25r34r24r25	ST398
S18	T730	r07r34r34r34r33r34	ST97
S15	T518	r04r20r17r23r24r20r17r25	ST50

### Biofilm Formation

All isolates had the ability to form biofilms. The rates of weak, moderate, and strong biofilm producers were 59.7% (37/62), 22.6% (14/62), and 17.7% (11/62), respectively (Table 2). Among the *S. aureus* isolates obtained from the Shandong herd, 27.8% (5/18) were weak biofilm producers, 38.9% (7/18) were moderate biofilm producers, and 33.3% (6/18) were strong biofilm producers. Most isolates of *S. aureus* from the Jiangsu herds showed a weak biofilm-forming ability, whereas, isolates obtained from the Guangdong herd were predominantly moderate biofilm producers.

### Antimicrobial Susceptibility

Antimicrobial resistance was most frequently observed to ciprofloxacin (29%,18/62), followed by penicillin (24.2%, 15/62), streptomycin (9.6%, 6/62), kanamycin (3.2%, 2/62), cephalexin (3.2%, 2/62), gentamicin (3.2%, 2/62), amikacin (3.2%, 2/62), and clindamycin (3.2%, 2/62). All isolates were sensitive to ceftiofur, tetracycline, and doxycycline (Table 4). Thirteen resistance patterns were identified and 11 isolates (17.7%, 11/62) were characterized as multidrug resistant (MDR) (Table 5). All MDR isolates were from the Jiangsu and Shandong herds. The relationship between prevalence of MDR strains and biofilm formation ability of the *S. aureus* isolates was further analyzed (Table 6). The proportion of MDR isolates was significantly higher in moderate biofilm producers than in weak biofilm producers (*p* < 0.05).

**Table 4:** Antimicrobial resistance of *S. aureus* isolates from different provinces.

		**Percentage (no.) of resistant isolates**			
**Antimicrobials**	**Zone diameter of resistance**	**Shandong (*n* = 18)**	**Jiangsu (*n* = 39)**	**Guangdong (*n* = 5)**	**Total (*n* = 62)**
Penicillin	≤ 28	33.3 (6)	23.1 (9)	0 (0)	24.2 (15)
Cephalexin	≤ 14	0 (0)	5.1 (2)	0 (0)	3.2 (2)
Ceftiofur	≤ 17	0 (0)	0 (0)	0 (0)	0 (0)
Gentamicin	≤ 12	5.6 (1)	2.5 (1)	0 (0)	3.2 (2)
Streptomycin	≤ 11	27.3 (5)	2.5(1)	0 (0)	9.6 (6)
Amikacin	≤ 14	0 (0)	5.1 (2)	0 (0)	3.2 (2)
Kanamycin	≤ 13	5.6 (1)	2.5 (1)	20 (1)	4.8 (3)
Tetracycline	≤ 14	0 (0)	0 (0)	0 (0)	0 (0)
Doxycycline	≤ 12	0 (0)	0 (0)	0 (0)	0 (0)
Ciprofloxacin	≤ 15	55.6 (10)	20.5 (8)	0 (0)	29.0 (18)
Clindamycin	≤ 14	11.1 (2)	0(0)	0 (0)	3.2 (2)

**Table 5:** The antimicrobial resistance patterns of 62 *S. aureus* isolates.

**Antimicrobial resistance patterns**	**Origin**	**No. of *S. aureus* isolates**
P	Jiangsu	1
STR	Jiangsu	1
CIP	Shandong	4
KAN	Guangdong	1
P-CIP	Jiangsu	3
STR-CIP	Shandong	1
P-AMI-CIP	Jiangsu	2
P-CEP-CIP	Jiangsu	2
P-KAN-CIP	Jiangsu	1
P-CN-CIP	Jiangsu	1
P-STR-CIP	Shandong	2
P-STR-CIP-DA	Shandong	2
P-CN-KAN-CIP	Shandong	1

**Table 6:** Association between multidrug resistance and biofilm formation.

**Phenotype**	**Percentage (no.) of** ***S. aureus*** **with biofilm production**		
	**Weak**	**Moderate**	**Strong**
Non-Multidrug resistance	91.9% (34)	64.3% (9)	72.7% (8)
Multidrug resistance	8.1% (3)	35.7% (5)^a^	27.3% (3)
Total	37	14	11

a *p < 0.05, the proportion of MDR strains were significantly higher in moderate biofilm producers than in weak biofilm producers*.

### Detection of Virulence Genes

The 12 virulence genes examined in this study were distributed with varying frequencies among the *S. aureus isolates* (Table 7). The genes *fnbA, clfB, icaA*, and *icaD* were detected in all isolates. Nearly all isolates also harbored the *hl*α (98.4%) and *hl*β (95.2%) genes. The *lukM, clfA, fnbB*, and *tsst-1* genes were detected in 71% (44/62), 50% (31/62), 21% (13/62), and 8.1% (5/62) of isolates, respectively. The genes *pvl* or *bap* were not detected in any of the isolates. Furthermore, 10 different virulence gene patterns were observed among the *S. aureus* isolates (Table 8). The most frequent number of virulence genes per isolate was 7 and this number was detected in all isolates.

**Table 7:** Virulence genes identification in *S. aureus* isolates from different provinces.

**Genes**	**Percentage (no.) of positive isolates**			
	**Guangdong (*n* = 5)**	**Jiangsu (*n* = 39)**	**Shandong (*n* = 18)**	**Total (*n* = 62)**
*fnbA*	100 (5)	100 (39)	100 (18)	100 (62)
*fnbB*	0 (0)	20.5 (8)	27.8 (5)	21 (13)
*clfA*	100 (5)	12.9 (8)	100 (18)	50 (31)
*clfB*	100 (5)	100 (39)	100 (18)	100 (62)
*icaA*	100 (5)	100 (39)	100 (18)	100 (62)
*icaD*	100 (5)	100 (39)	100 (18)	100 (62)
*bap*	0 (0)	0 (0)	0 (0)	0 (0)
*hlα*	100 (5)	97.4 (38)	100 (18)	98.4 (61)
*hlβ*	100 (5)	94.9 (37)	94.4 (17)	95.2 (59)
*lukM*	100 (5)	94.9 (37)	11.11 (2)	71 (44)
*tsst-1*	80 (4)	0 (0)	5.6 (1)	8.1 (5)
*pvl*	0 (0)	0 (0)	0 (0)	0 (0)

**Table 8:** The virulence gene patterns of 62 *S. aureus* isolates.

**Virulence gene patterns**	**No. of *S. aureus***
*hlβ-fnbA-fnbB-clfA-clfB-icaA-icaD*	1
*lukM-hlα-fnbA-fnbB-clfA-clfB-icaA-icaD*	2
*lukM-hlα-hlβ-fnbA-fnbB-clfA-clfB-icaA-icaD*	4
*hlα-hlβ-fnbA-fnbB-clfA-clfB-icaA-icaD*	5
*hlα-hlβ-fnbA-fnbB-clfA-clfB-icaA-icaD-tsst-1*	1
*lukM-hlα-hlβ-fnbA-clfB-icaA-icaD*	31
*hlα-hlβ-fnbA-clfA-clfB-icaA-icaD*	10
*lukM-hlα-hlβ -fnbA-clfA-clfB-icaA-icaD-tsst-1*	4
*lukM-hlα-hlβ-fnbA-clfA-clfB-icaA-icaD*	3
*hlα-fnbA-clfA-clfB-icaA-icaD*	1

## Discussion

Mastitis is an important economic disease restricting the development of the dairy industry. Rapid and accurate identification of pathogens is crucial for the development of targeted prevention and control strategies for mastitis, especially on large-scale farms. MALDI-TOF MS is a rapid and reliable technique for the accurate detection of various microorganisms (22). In this study, 62 *S. aureus* isolates were identified in milk samples from clinical bovine mastitis cases and were subjected to cluster analysis by MALDI-TOF MS. This is the first time that this technique has been applied to detect and analyze bovine mastitis pathogens in China. The incidence of clinical mastitis caused by *S. aureus* was 9.9% in this investigation, similar to the results of Gao et al. (8). In contrast, a lower incidence (2.8%) of *S. aureus* bovine mastitis was reported in large dairy herds in Wisconsin, USA (23). The emergence of MRSA not only challenges the treatment of bovine mastitis, but also poses a threat to human health through food chain or other ways. In this study, eight of the 62 isolates were OS-MRSA. A high prevalence of OS-MRSA has been reported among *S. aureus* of bovine mastitis origin in China (24). Molecular typing indicated that eight OS-MRSA isolates belonged to t2196-*agr* I and the genotype pattern of the representative MRSA isolate was ST630-CC8-t2196-*agr* I. Previous studies revealed that the *spa* types of OS-MRSA from different regions were diverse. The *spa* types t267, t1234, t324, and t121 were found in OS-MRSA isolates from different countries (24, 25).

The diverse genetic backgrounds of the 62 isolates were indicated by *agr* typing, *spa* typing, and MLST. The predominant *agr* type in this study was *agr* II. This was in contrast to previous reports, in which *agr* I was the dominant type in *S. aureus* isolated from bovine mastitis cases (26, 27). Strains of *S. aureus* belonging to *agr* type I were reported to invade epithelial cells more efficiently than strains classified into other *agr* types (28). This is more likely to result in antibiotic treatment failure. A certain proportion of *S. aureus* isolates from different farms in the current study were classified as *agr* I type. In terms of *spa* types, seven recognized *spa* types were identified among the 62 isolates. The most common *spa* type was t529, consistent with a previously report in which t529 was the dominant *spa* type in Switzerland. Isolates shown to belong to *agr* II has only a single *spa* type, however, there were seven or four *spa* types among the isolates belonging to *agr* I or *agr* IV. *Seven* representative isolates were selected from different *spa* types for MLST typing. ST97 and ST50 each corresponded to two *spa* types (ST97-t2734 and ST97-t730, and ST50-t518 and ST50-t571, respectively). Another isolate was identified as ST398, which was common livestock-associated MRSA type (29). The ST398 was originally reported in swine farmers and often appeared in Europe and America countries (30). Furthermore, ST398 MRSA was shown to correspond to several distinct *spa* types (t011, t034, t108, and t1451) and was resistant to many non-β-lactam antibiotics (31). In this study, an isolate of ST398-t034 was MSSA and was sensitive to the tested antibiotics. To further understand the circulation of *S. aureus*, cluster analysis based on MALDI-TOF MS spectra was performed. In group F, the isolates obtained from different farms were classified into sub-clades at a close relative distance, which indicated that there were some relationships in the circulation of *S. aureus* between the different herds. The development of the dairy industry in China has led to many small farms bring eliminated or choosing to expand, resulting in cows being transferred between different regions or farms. This transfer of animals provides opportunities for *S. aureus* to spread between different herds.

Information concerning the antimicrobial resistance of pathogens is crucial for selecting effective antibiotic therapies. In this study, antimicrobial resistance to ciprofloxacin was most frequently observed. Previous studies reported that antimicrobial resistance to ciprofloxacin ranged from 29.6 to 53.4% in *S. aureus* isolated from bovine mastitis in China (29, 32, 33). Conversely, ciprofloxacin-resistant *S. aureus* is rarely detected in other countries (34, 35). Fluoroquinolone antibiotics are regarded as extremely important antibiotics for human use by the World Health Organization (36). Consequently, special care should be taken regarding the use of these antibiotics in livestock. *S. aureus* was found to be the most common penicillin-resistant pathogen in bovine mastitis (37). In this investigation, penicillin resistance was the second most prevalent antimicrobial resistance phenotype. The proportion of clinical mastitis caused by *S. aureus* varies greatly (from 3.6 to 24.2%) on the studied farms. The high proportion of *S. aureus* in Jiangsu herds was speculated to be related to the use of benzylpenicillin, nafcillin sodium, and dihydrostreptomycin sulfate in dry cows. Because some strains of *S. aureus* isolated from the Jiangsu herds were resistant to penicillin and streptomycin. The emergence of MDR *S. aureus* has become a growing public health concern. In this study, 54.5% (6/11) of MDR *S. aureus* isolates belong to *spa* type t2196-*agr* I. *Staphylococcal* biofilms can enhance the resistance of *S. aureus* to antibiotics (38). In this investigation, the percentage of MDR isolates was significantly higher in moderate biofilm producers than in weak biofilm producers. In contrast, Zhang et al. (39) reported that there was no significant association between MDR and biofilm formation ability of *S. aureus* isolated from pork production samples.

Adhesion were essential for *S. aureus* to invade host cells and evade immune responses (40). Specific genetic changes in *spa, clfA, fnbA*, as well as a loss of *fnbB* can lead to a Staphaurex-negative phenotype of *S. aureus* (41). In this study, all *S. aureus* isolates harbored the *fnbA* and *clfB* genes. This was congruent with previous reports, in which *fnbA* and *clfB* were detected in all *S. aureus* isolates from cases of bovine mastitis (21, 29). In contrast, only 21% of isolates harbored the *fnbB* gene, which was much lower than that of previous reports (40, 42). Similarly, Gogoi et al. (43) reported that the *fnbB* gene was only detected in 1.3% of *S. aureus* isolates in Australia. Biofilm contributes to the development of antimicrobial resistance in *S. aureus*. All isolates in the current study had the ability to form biofilm. Biofilm-related genes (*icaA* and *icaD*) were detected in all isolates, and none of the isolates harbored biofilm associated protein (*bap*) gene. This was in agreement with previous reports, in which the genes *icaA* and *icaD* were frequently detected, but the *bap* gene was low incidence or not detected in *S. aureus* of bovine mastitis origin (12, 29, 40). The high prevalence of *hl*α and *hl*β in this study was similar to previous reports in South Africa (100%) and in other regions of China (94.3 and 97.1%, respectively) (26, 42). Furthermore, 16 *hlb*-positive isolates failed to produce β-hemolysin though Christie, Atkins, and Munch-Petersen (CAMP) tests (data not shown). The reason for this may be associated with the *sak* gene encoding staphylokinase, which can be integrated into the *hlb* gene by phage and causes *S. aureus* to fail to produce β-hemolysin (44). *S. aureus* can acquire two phage-encoded leukocidins, Panton-Valentine leukocidin (PVL) and LukMF. None of the tested isolates in this study harbored the *pvl* genes, and this was in agreement with previous investigations, where the *pvl* gene were often absent in *S. aureus* isolated from bovine mastitis (45, 46). The bicomponent leukocidin LukMF has high leukotoxic activity to bovine polymorphonuclear cells (47) and the level of LukM in milk is associated with the severity of mastitis during the course of infection (48). The genes encoding this leukocidin are predominantly found in *S. aureus* isolated from ruminants with mastitis (49). The high prevalence of the *lukM* gene in the current study was in agreement with other reports (50, 51). Another toxin, Tsst-1 causes toxic shock syndrome in humans by hyperactivation the host immune response (52). In this study, five *S. aureus* isolates harbored the *tsst-1* gene. It is imperative for the protection of public health to continuously monitor the epidemiology of such super antigenic toxin genes.

In conclusion, this study presents the first insights into the characterization of *S. aureus* isolated from clinical mastitis cases on large-scale dairy farms in China. The findings suggest that selecting effective antibiotics is important to reduce the incidence of *S. aureus* mastitis in dry cows. Moreover, it is necessary to strictly implement inspection and quarantine procedures to prevent the transmission of *S. aureus* among different farms. Knowledge generated in this study will contribute to improvements in prevention and control strategies to minimize the risk of bovine mastitis and associated losses on the large-scale dairy farms.

## Data Availability Statement

The original contributions presented in the study are included in the article/supplementary material, further inquiries can be directed to the corresponding author/s.

## Ethics Statement

The animal study was reviewed and approved by the departmental committee of the College of Veterinary Medicine, Yangzhou University.

## Author Contributions

HW participated in the design of this study. KL, LT, LF, JuL, and CB collected the samples. KL and LT performed the experiments. KL analyzed the data and wrote the paper. JiL, GZ, LC, and XM contributed to the preparation of the manuscript. All authors read and approved the final version of the manuscript.

## Conflict of Interest

The authors declare that the research was conducted in the absence of any commercial or financial relationships that could be construed as a potential conflict of interest.
